# Profiling interactions of vaborbactam with metallo-β-lactamases

**DOI:** 10.1016/j.bmcl.2019.05.031

**Published:** 2019-08-01

**Authors:** Gareth W. Langley, Ricky Cain, Jonathan M. Tyrrell, Philip Hinchliffe, Karina Calvopiña, Catherine L. Tooke, Emma Widlake, Christopher G. Dowson, James Spencer, Timothy R. Walsh, Christopher J. Schofield, Jürgen Brem

**Affiliations:** a Department of Chemistry, University of Oxford, Chemistry Research Laboratory, 12 Mansfield Road, Oxford OX1 3TA, United Kingdom; b Current address: Charles River Laboratories, Chesterford Research Park, Saffron Walden, Essex CB10 1XL, United Kingdom; c School of Life Sciences, Gibbet Hill Campus, University of Warwick, Coventry CV4 7AL, United Kingdom; d Department of Medical Microbiology & Infectious Disease, Institute of Infection & Immunity, UHW Main Building, Heath Park, Cardiff CF14 4XN, United Kingdom; e School of Cellular and Molecular Medicine, Biomedical Sciences Building, University of Bristol, Bristol BS8 1TD, United Kingdom

**Keywords:** Vaborbactam, Serine- and metallo-β-lactamase, Transition state analogue, Boronate inhibitor, β-Lactamase induction, Antibiotic resistance

## Abstract

β-Lactams are the most successful antibacterials, yet their use is threatened by resistance, importantly as caused by β-lactamases. β-Lactamases fall into two mechanistic groups: the serine β-lactamases that utilise a covalent acyl-enzyme mechanism and the metallo β-lactamases that utilise a zinc-bound water nucleophile. Achieving simultaneous inhibition of both β-lactamase classes remains a challenge in the field. Vaborbactam is a boronate-based inhibitor that reacts with serine-β-lactamases to form covalent complexes that mimic tetrahedral intermediates in catalysis. Vaborbactam has recently been approved for clinical use in combination with the carbapenem meropenem. Here we show that vaborbactam moderately inhibits metallo-β-lactamases from all 3 subclasses (B1, B2 and B3), with a potency of around 20–100 fold below that by which it inhibits its current clinical targets, the Class A serine β-lactamases. This result contrasts with recent investigations of bicyclic boronate inhibitors, which potently inhibit subclass B1 MBLs but which presently lack activity against B2 and B3 enzymes. These findings indicate that cyclic boronate scaffolds have the potential to inhibit the full range of β-lactamases and justify further work on the development of boronates as broad-spectrum β-lactamase inhibitors.

## Graphical abstract

**Figure:**
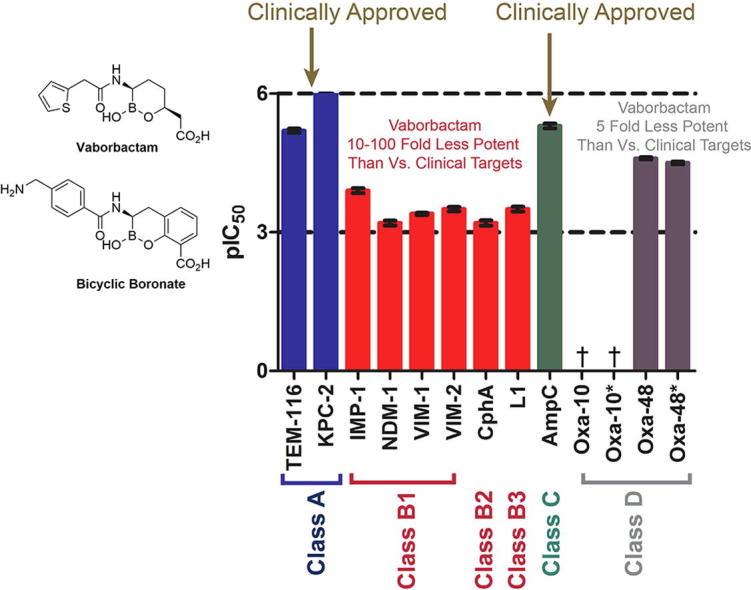

The β-lactams are amongst the most important antibacterials;^1^ their continued widespread use is challenged by resistance, most importantly due to β-lactamases.^2^ There are two mechanistically distinct types of β-lactamases: the serine–β-lactamases (SBLs; Ambler classes A, C and D)^3^ and the metallo–β-lactamases (MBLs; class B)^4^ (Fig. 1). SBL inhibitors (clavulanate, sulbactam and tazobactam) are established for clinical use when combined with a penicillin/cephalosporin.^5^ In combination with a cephalosporin, the non β-lactam SBL inhibitor Avibactam has been introduced as a broader–spectrum SBL inhibitor (active against classes A and C, with limited activity against class D).^6^, ^7^, ^8^ None of the clinically used SBL inhibitors inhibit MBLs. The β-lactams of the established SBL inhibitors are also increasingly subject to hydrolysis by MBLs/SBLs^7^, ^8^ and even the cyclic urea core of avibactam is susceptible to low-level hydrolysis by some MBLs.^9^ There is thus increasing interest in developing non-hydrolytically labile β-lactamase inhibitors.^10^ In this regard, boronic acids have long attracted attention since they can mimic the tetrahedral intermediates common to SBL and MBL catalysis (Fig. 1).^11^

**Figure 1:**
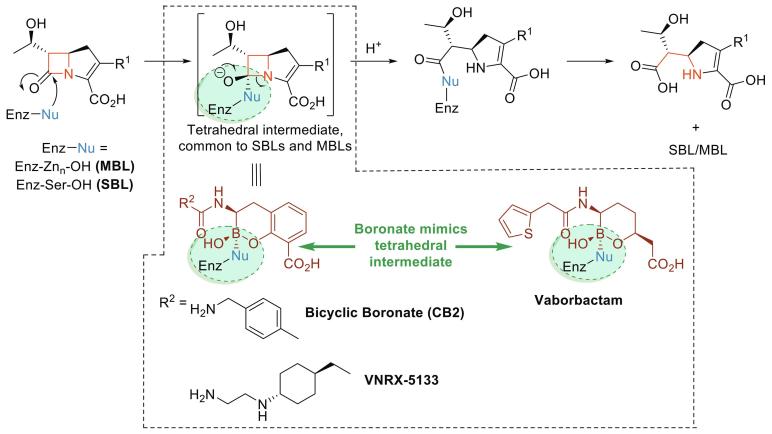
Outline mechanism of β-lactamase catalysis exemplified for a carbapenem. Note that the product can be produced in different tautomeric forms. The tetrahedral intermediate, common to both SBLs and MBLs, is mimicked by cyclic boronates.

Vaborbactam (formerly RPX7009) was developed to target SBLs of classes A and C^12^ and has been recently approved for clinical use in combination with meropenem (Vabomere).^13^, ^14^ In an initial study,^12^ vaborbactam was described as a sub-micromolar inhibitor of clinically relevant SBLs, with K_i_ values (using nitrocefin assays) for SBLs, including extended spectrum-β-lactamases (ESBLs), in the 10–100 nM range (CTX-M15 K_i_ 44 nM; SHV-12 K_i_ 29 nM; TEM-10 K_i_ 110 nM; KPC-2 carbapenemase K_i_ = 69 nM (all class A); *Enterobacter cloacae* cephalosporinase P99 K_i_ = 53 nM; *Klebsiella pneumonia*^15^ CMY-2 K_i_ 99 nM (class C)).^12^ A subsequent study reported that vaborbactam inhibition manifests fast-on-fast-off behaviour, a feature proposed to underlie lack of potent inhibition of the SHV-12 SBLs (and the TEM-42 ESBL).^13^ Co-administration of vaborbactam with a β-lactam antibiotic (primarily meropenem) manifests activity against bacterial strains harbouring genes encoding diverse class A enzymes (TEM-116; CTX-M, SHV, and TEM ESBLs; the KPC, FRI-1 and SME-2 carbapenemases) and the narrow spectrum oxacillinases OXA-2 and OXA-30.^12^ By contrast, vaborbactam combinations were not active against strains harbouring OXA-48 like class D SBLs, those hyperexpressing chromosomally encoded AmpC SBLs, and/or producing MBLs (i.e. the NDM, IMP or VIM carbapenemases).^12^, ^13^, ^16^, ^17^ Boronates with a ‘bicyclic’ scaffold such as cyclic boronate CB2^11^, ^18^ (Fig. 1) can inhibit all four Ambler classes, with one such compound, VNRX-5133, in clinical trials^11^, ^15^, ^18^ (Fig. 1). By contrast, vaborbactam, which is principally ‘monocyclic’ in solution (Fig. 1), is reported not to inhibit MBLs.^12^, ^13^, ^14^

Here we report studies profiling the interactions of vaborbactam with representative enzymes of the three MBL subclasses (B1, B2, B3). The results reveal that vaborbactam shows weak inhibition activity of all three MBLs subclasses, including the clinically relevant B1 MBLs Verona Integron-encoded MBL (VIM)-1 and VIM-2, the New Delhi MBL (NDM)-1 and Imipenemase (IMP)-1; the B2 MBL *Aeromonas hydrophila* CphA (CphA) and the B3 MBL L1 from *Stenotrophomonas maltophilia*.

As anticipated, based on prior reports,^12^, ^13^, ^17^ vaborbactam inhibits representative SBLs from classes A and C, i.e. the class A narrow spectrum β-lactamase TEM-116 (IC_50_ = 6 μM), the Class A carbapenemase KPC-2 (IC_50_ = 90 nM), and the class C cephalosporinase AmpC from *Pseudomonas aeruginosa* (IC_50_ = 5 μM) (Table 1). Against the tested class D enzymes, moderate inhibition of the OXA-48 carbapenemase was observed (IC_50_ = 25 μM and IC_50_ = 32 μM in the presence of 100 mM NaHCO_3_), whilst only very low-level inhibition (<50%) of the narrow spectrum oxacillinase OXA-10 was observed using 400 μM vaborbactam (Table 1).

**Table 1:** IC_50_ values and reported K_i_ values for vaborbactam against β-lactamases, compared to the reported values for vaborbactam and a bicyclic boronate.^18^^†^Weak inhibition (<50%) was observed for OXA-10 at the highest tested concentration (400 μM).

Class	Enzyme	Vaborbactam IC_50_ [μM]	Vaborbactam K_i_ [nM]	Cyclic Boronate (CB2)^11^, ^18^ IC_50_ [μM]
A	TEM-116	6 μM	Not available	0.003 μM^18^
A	CTX-M15	Not available	44 nM^12^	0.013 μM^11^
A	SHV-12	Not available	29 nM^12^	Not available
A	TEM-10	Not available	110 nM^12^	Not available
A	KPC-2	0.09 μM	69 nM^12^	0.03 μM
B1	IMP-1	126 μM	Not available	1 μM^18^
	NDM-1	631 μM	Not available	0.029 μM^18^
	VIM-1	398 μM	Not available	0.085 μM^11^
	VIM-2	316 μM	Not available	0.003 μM^18^
B2	CphA	631 μM	Not available	> 100 μM^18^
B3	L1	336 μM	Not available	Not inhibited^19^
C	AmpC	5 μM	Not available	0.12 μM^11^
C	P99	Not available	53 nM^12^	Not available
C	CMY-2	Not available	99 nM^12^	Not available
D	OXA-10	> 400 μM	Not available	Not available
	OXA-10	> 400 μM	Not available	5.1 μM^11^
	(100 mM NaHCO_3_)			
	OXA-48	25 μM	Not available	Not available
	OXA-48	32 μM	Not available	2.6 μM^11^
	(100 mM NaHCO_3_)			

Vaborbactam was then tested against a panel of MBLs (subclass B1: IMP-1, VIM-1, VIM-2, NDM-1; subclass B2: CphA and subclass B3: L1) comprising representatives of the three MBL subclasses (which differ in their active site architectures and Zn(II) requirements).^18^, ^20^, ^21^ Vaborbactam weakly inhibits all four of the tested B1 MBLs, VIM–1 (IC_50_ = 398 μM), VIM-2 (IC_50_ = 316 μM), NDM-1 (IC_50_ = 631 μM) and IMP-1 (IC_50_ = 126 μM), but at a much lower levels than observed for the SBLs. Similar low–level inhibition of the MBL subclass B2 CphA (IC_50_ = 631 μM) and the subclass B3 L1 (IC_50_ = 336 μM) was also observed (Table 1).

We investigated the antimicrobial activity of vaborbactam at a fixed concentration of 8 µg/mL (27 µM), in combination with meropenem against three *E. coli* and *K. pneumoniae* clinical isolates all co-expressing NDM-1, which is weakly inhibited by vaborbactam (IC_50_ = 631 μM). In accord with the literature data^12^, ^13^ and its relatively weak potency versus NDM-1 vaborbactam did not improve the MIC of meropenem against these strains (Supporting Information- Table 1.).

Although there are multiple crystal structures of boronates complexed to both SBLs^22^ and the related penicillin binding proteins,^23^ there are few with MBLs.^11^, ^18^ To investigate the possible structural basis of vaborbactam interaction with the MBLs, a model of vaborbactam bound to the B1 MBL VIM-2, based upon the binding mode of a bicyclic boronate (Fig. 1, PDB ID: 5FQC),^18^ was constructed (Fig. 2C).

**Figure 2:**
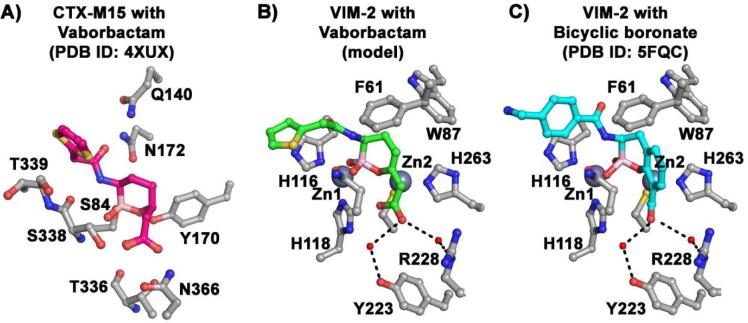
Model of vaborbactam binding to VIM-2 (B). Residues within 3.5 Å of vaborbactam are indicated. The model is presented alongside a view from a crystal structure of (A) vaborbactam bound to CTX-M15 (PDB ID: 4XUX) and (C) a bicyclic boronate bound to VIM-2 (PDB ID: 5FQC).^18^

The model implies that vaborbactam might bind in a similar manner to the bicyclic boronates (Fig. 2B and C),^11^, ^24^ with its ‘endocyclic’ boronate oxygen positioned to bind to the Zn(2) ion in the Cys-His-Asp site of the di-Zn(II) active site and the other two boronate oxygens positioned to bind to Zn(1) in the tri-His site. Since VIM-1 and VIM-2 employ different binding modes for the substrate carboxylate (VIM-2: Y224, R228 and VIM-1: H224,S228),^24^ the observation that VIM-1 and VIM-2 are inhibited to a similar degree by vaborbactam is notable. The modelled VIM-2 complex features water-mediated contacts between the vaborbactam carboxylate and Y224 and R228, as observed in our previous crystallographic characterisations of bicyclic boronate binding to MBLs^18^ (Fig. 2B and C).

The overall results reveal that, from comparison of IC_50_ values, vaborbactam manifests inhibition of SBLs (TEM-116, KPC-2 and AmpC, from classes A and C, respectively) that is 20 to 7000–fold more potent than that for the class B MBLs (IMP-1, VIM-1, VIM-2, NDM-1 and L1) and 5-fold more potent than reported for the class D SBL, OXA-48 (Table 1). With the class D enzymes (OXA-10 and OXA-48) vaborbactam manifests weak activity against the carbapenem hydrolysing class D (CHDL) SBL OXA-48, but no activity against the narrow spectrum oxacillinase OXA-10 (Fig. 3). These observations correlate with microbiological studies, wherein vaborbactam shows no activity against OXA-10/OXA–48^17^ and as reported here, NDM-1 producing strains. Although of weak potency against MBLs, vaborbactam exhibits greater activity against the MBLs than avibactam, which we have demonstrated to interact with some MBLs^9^ but which does not show any inhibition across the same range of inhibitor concentrations. Notably, vaborbactam shows some activity towards the (mono-Zn(II)) B2 MBL CphA and the B3 MBL L1 (Table 1). For the class B1 MBLs, vaborbactam was most potent against IMP-1 (126 µM), and less potent against VIM-1 and VIM-2 (398 and 316 µM, respectively) with the lowest activity observed against NDM-1 (631 µM).

**Figure 3:**
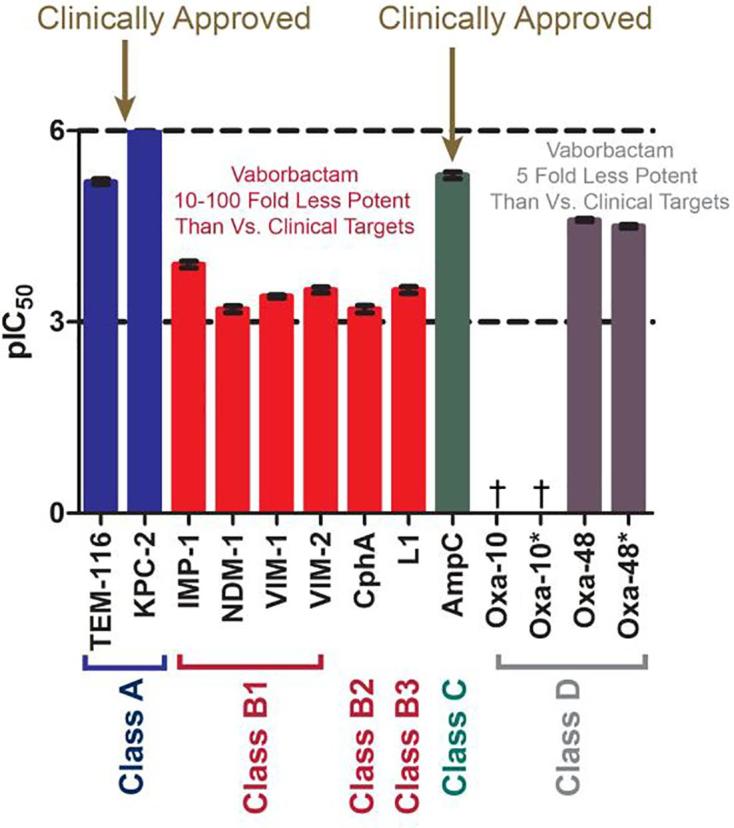
IC_50_ values for vaborbactam against the shown β-lactamases.

The results imply that whilst vaborbactam itself is very likely not useful against most, if not all, MBL-, and many SBL- (especially class D), producing strains, there is considerable potential for further optimisation of cyclic boronate based β -lactamase inhibitors. Boronates are being pursued as SBL/MBL/penicillin-binding protein (PBP) inhibitors, in part because of their ability to mimic potentially common tetrahedral intermediates in catalysis.^12^, ^18^ However, while such efforts are currently limited by the lack of useful (broad spectrum and potent) PBP inhibition by the boronates so far investigated, structure-activity relationship (SAR) information is emerging for SBL/MBL inhibition by different types of boronates. By contrast to the results for the monocyclic compound vaborbactam reported here, bicyclic boronates are capable of potent (nM) inhibition of MBLs of subclass B1 in addition to their activity against SBLs. However, the currently tested bicyclic boronates e.g. CB2, Table 1,^18^, ^19^ do not exhibit inhibitory activity against the B2 CphA (mono Zn(II)) or B3 L1 MBLs. It is notable that vaborbactam shows weak but detectable (μM) inhibition of both CphA and L1, raising the possibility that monocyclic boronates are potentially capable of supporting broader spectrum inhibitory activity against MBLs than their current bicyclic counterparts. Together with previous studies, including those with PBPs,^23^ these observations may reflect the increased conformational flexibility of monocyclic versus bicyclic boronates and, maybe, the increased propensity of the former to exist in an acyclic form. Further SAR on both mono- and bi-cyclic boronate based β-lactamase inhibitors is required.

We also observed substantial variations in vaborbactam potency within, as well as between, different MBL subclasses (B1-B3). The differences in vaborbactam activity against B1 MBLs (IMP-1 > VIM-1/VIM-2 > NDM-1), might relate to the active site of IMP-1 being more compact (on the basis of reported crystallographic studies) than that of NDM-1;^25^ bicyclic boronates inhibit IMP-1 less potently than VIM-1/-2 and NDM-1.^11^ For the class D enzymes, which require active site lysine carbamylation for activity;^26^ vaborbactam inhibition was unaffected by addition of NaHCO_3_ to the assay buffer, although this increased catalytic activity. This observation is consistent with reported studies on bicyclic boronates,^18^ but contrasts with results for avibactam.^26^ The molecular reasons for these variations in SAR for the different classes of boronate based inhibitors are presently unclear, but merit further detailed investigation given the desirability of developing very broad spectrum β–lactamase inhibitors, especially those active against carbapenemases, e.g. the VIM, IMP, NDM and OXA-48 enzymes, for which current inhibitors are largely ineffective.

Overall, our results identify vaborbactam as a low level pan β-lactamase inhibitor able to inhibit SBLs and MBLs of all classes. Together with recently reported studies on the structural bases of (bi)cyclic boronate inhibition of all classes of β-lactamases and PBPs, these data support the proposal that cyclic boronates constitute inhibitor templates of interest for development as β -lactamase inhibitors with wider spectra of activity than currently available agents.
