# Supplementary material for: Profiling interactions of vaborbactam with metallo-β-lactamases
Source: Bioorg Med Chem Lett. 2019 Aug 1;29(15):1981–4. doi: 10.1016/j.bmcl.2019.05.031 (PMC6593178; doi:10.1016/j.bmcl.2019.05.031)
Supplement: Supplementary data 1 [file mmc1.docx]

**Electronic Supplementary Information**

**Profiling Interactions of Vaborbactam with Metallo-β-Lactamases**

Gareth W. Langley^a,b^, Ricky Cain^c^, Jonathan M. Tyrrell^d^, Philip Hinchliffe^e^, Karina Calvopiña^a^, Catherine L. Tooke^e^, Emma Widlake^d^, Christopher G. Dowson^c^, James Spencer^e^, Timothy R. Walsh^d^, Christopher J. Schofield^a*^, and Jürgen Brem^a*^.

^a^Department of Chemistry, University of Oxford, Chemistry Research Laboratory, 12 Mansfield Road, Oxford, OX1 3TA, United Kingdom.

^b^Current Address: Charles River Laboratories, Chesterford Research Park, Saffron Walden, Essex, CB10 1XL, United Kingdom.

^c^School of Life Sciences, Gibbet Hill Campus, University of Warwick, Coventry, CV4 7AL, United Kingdom.

^d^Department of Medical Microbiology & Infectious Disease, Institute of Infection & Immunity, UHW Main Building, Heath Park, Cardiff, CF14 4XN, United Kingdom.

^e^School of Cellular and Molecular Medicine, Biomedical Sciences Building, University of Bristol, Bristol, BS8 1TD, United Kingdom.

*Address correspondence:

Prof. Christopher Schofield; Tel: +44 (0)1865 275625; Fax: +44 (0)1865 285002; Email: christopher.schofield@chem.ox.ac.uk, or Dr. Jürgen Brem; Email: jurgen.brem@chem.ox.ac.uk.

**Materials and Methods**

**Enzyme production and assays**

Recombinant MBLs and SBLs were produced in *E. coli* and purified following reported procedures^1-6^ and were all >95% pure (as judged by SDS-PAGE and MS analyses). Note the TEM enzyme used in this study is named TEM-116^7^, in previous studies this has been assigned incorrectly as TEM-1^3, 5, 8-14^. The inhibition activity of vaborbactam was tested using a panel of recombinant enzymes covering all four Ambler classes of β-lactamase (A, B, C and D), including representatives from all three subclasses of MBL (*i.e.* enzymes from classes B1, B2 and B3). Activity was assayed by monitoring the release of a fluorophore following the enzymatic breakdown of the cephalosporin FC-5^15^, with the exception of the class B2 MBLs, where the hydrolysis of meropenem was used as a readout^16^. FC-5 assays were conducted in clear bottomed 384 black well microplates (Greiner) and the initial rate of reaction was assessed by monitoring the fluorescence intensity (λ_ex_ = 380 nm and λ_em_ = 460 nm) using a PHERAstar FS (BMG LabTech) plate reader^15^. For the B2 MBLs, assays were carried out in 96 well UV star microplates (Greiner) and UV absorbance at λ = 300 nm monitored. Initial rates of reaction were determined and dose-response analyses conducted in GraphPad Prism^15^.

**Binding mode of vaborbactam**

To investigate the structural basis of vaborbactam interaction with the MBLs, a model of vaborbactam with the B1 MBL VIM-2, based upon the binding mode of a bicyclic boronate (PDB ID: 5FQC) was constructed. Studies were conducted using AutoDock 4.21 (http://autodock.scripps.edu) and the active site was defined using Maestro (https://www.schrodinger.com/maestro). The *in silico* docking studies conducted using AutoDock 4.21 were performed as described^2^.

**Antimicrobial Susceptibility Testing**

Minimal inhibitory concentrations (MICs) for Meropenem (MEM) alone (0.06-64 µg mL^-1^) and in combination with vaborbactam (8 µg mL^-1^) were evaluated against a small collection of clinical, carbapenemase-producing *Escherichia coli and Klebsiella pneumoniae* strains. MICs were determined by the broth dilution method, and interpreted using published guidelines described by EUCAST/CLSI^17^.

**Supplementary Information Table 1.** In vitro cell-based screening of Vaborbactam.

| **Strain** | **Species** | **Genotype** | **Meropenem** | **Meropenem**  **+**  **Vaborbactam** |
| --- | --- | --- | --- | --- |
| **S117** | ***E. coli*** | **NDM-1** | **>64** | **>64** |
| **IR57** | ***E. coli*** | **NDM-1** | **>64** | **>64** |
| **B64** | ***K. pneumoniae*** | **NDM-1** | **>64** | **>64** |
| **B68-1** | ***K. pneumoniae*** | **NDM-1** | **>64** | **>64** |
| **IR43** | ***K. pneumoniae*** | **NDM-1** | **>64** | **>64** |
| **91N** | ***E. coli*** | **NDM-1** | **>64** | **>64** |

**References**

1. Calvopina K, Hinchliffe P, Brem J, et al. Structural/mechanistic insights into the efficacy of nonclassical β-lactamase inhibitors against extensively drug resistant Stenotrophomonas maltophilia clinical isolates. *Mol Microbiol,* 2017;106(3): 492-504.

2. Cahill ST, Tyrrell JM, Navratilova IH, et al. Studies on the inhibition of AmpC and other β-lactamases by cyclic boronates. *Biochim Biophys Acta Gen Subj,* 2019;1863(4): 742-748.

3. Cahill ST, Cain R, Wang DY, et al. Cyclic Boronates Inhibit All Classes of β-Lactamases. *Antimicrob Agents Chemother,* 2017;61(4).

4. Brem J, van Berkel SS, Zollman D, et al. Structural Basis of Metallo-β-Lactamase Inhibition by Captopril Stereoisomers. *Antimicrob Agents Chemother,* 2015;60(1): 142-150.

5. Brem J, Cain R, Cahill S, et al. Structural basis of metallo-β-lactamase, serine-β-lactamase and penicillin-binding protein inhibition by cyclic boronates. *Nat Commun,* 2016;7: 12406.

6. Lohans CT, Chan HTH, Malla TR, et al. Non-Hydrolytic β-Lactam Antibiotic Fragmentation by L,D-Transpeptidases and Serine β-Lactamase Cysteine Variants. *Angew Chem Int Ed,* 2019;58(7): 1990-1994.

7. Jacoby GA, Bush K. The Curious Case of TEM-116. *Antimicrob Agents Chemother,* 2016;60(11): 7000-7000.

8. Inglis SR, Strieker M, Rydzik AM, Dessen A, Schofield CJ. A boronic-acid-based probe for fluorescence polarization assays with penicillin binding proteins and β-lactamases. *Anal Biochem,* 2012;420(1): 41-47.

9. Brem J, van Berkel SS, Aik W, et al. Rhodanine hydrolysis leads to potent thioenolate mediated metallo-β-lactamase inhibition. *Nat Chem,* 2014;6(12): 1084-1090.

10. Abboud MI, Damblon C, Brem J, et al. Interaction of Avibactam with Class B Metallo-β-Lactamases. *Antimicrob Agents Chemother,* 2016;60(10): 5655-5662.

11. Li GB, Abboud MI, Brem J, et al. NMR-filtered virtual screening leads to non-metal chelating metallo-β-lactamase inhibitors. *Chem Sci,* 2017;8(2): 928-937.

12. Lohans CT, Brem J, Schofield CJ. New Delhi Metallo-β-Lactamase 1 Catalyzes Avibactam and Aztreonam Hydrolysis. *Antimicrob Agents Chemother,* 2017;61(12): e01224-01217.

13. Cain R, Brem J, Zollman D, et al. In Silico Fragment-Based Design Identifies Subfamily B1 Metallo-β-lactamase Inhibitors. *J Med Chem,* 2018;61(3): 1255-1260.

14. Zhang D, Markoulides MS, Stepanovs D, et al. Structure activity relationship studies on rhodanines and derived enethiol inhibitors of metallo-β-lactamases. *Bioorg Med Chem Lett,* 2018;26(11): 2928-2936.

15. van Berkel SS, Brem J, Rydzik AM, et al. Assay platform for clinically relevant metallo-β-lactamases. *J Med Chem,* 2013;56(17): 6945-6953.

16. Makena A, Brem J, Pfeffer I, et al. Biochemical characterization of New Delhi metallo-beta-lactamase variants reveals differences in protein stability. *J Antimicrob Chemother,* 2015;70(2): 463-469.

17. Wayne PA. *Performance standards for antimicrobial susceptibility testing, 27th ed. CLSI supplement M100*: Clinical and Laboratory Standards Institute; 2017.
